# Screening for Genes Coding for Putative Antitumor Compounds, Antimicrobial and Enzymatic Activities from Haloalkalitolerant and Haloalkaliphilic Bacteria Strains of Algerian Sahara Soils

**DOI:** 10.1155/2014/317524

**Published:** 2014-05-27

**Authors:** Okba Selama, Gregory C. A. Amos, Zahia Djenane, Chiara Borsetto, Rabah Forar Laidi, David Porter, Farida Nateche, Elizabeth M. H. Wellington, Hocine Hacène

**Affiliations:** ^1^Microbiology Group, Laboratory of Cellular and Molecular Biology, Faculty of Biological Sciences, USTHB, BP 32, EL ALIA, Bab Ezzouar, Algiers, Algeria; ^2^School of Life Sciences, University of Warwick, Coventry CV4 7AL, UK; ^3^Department de Biologie, Ecole Normale Superieure (ENS), Vieux Kouba, Alger, Algeria

## Abstract

Extreme environments may often contain unusual bacterial groups whose physiology is distinct from those of normal environments. To satisfy the need for new bioactive pharmaceuticals compounds and enzymes, we report here the isolation of novel bacteria from an extreme environment. Thirteen selected haloalkalitolerant and haloalkaliphilic bacteria were isolated from Algerian Sahara Desert soils. These isolates were screened for the presence of genes coding for putative antitumor compounds using PCR based methods. Enzymatic, antibacterial, and antifungal activities were determined by using cultural dependant methods. Several of these isolates are typical of desert and alkaline saline soils, but, in addition, we report for the first time the presence of a potential new member of the genus *Nocardia* with particular activity against the yeast *Saccharomyces cerevisiae*. In addition to their haloalkali character, the presence of genes coding for putative antitumor compounds, combined with the antimicrobial activity against a broad range of indicator strains and their enzymatic potential, makes them suitable for biotechnology applications.

## 1. Introduction

There is an increasingly urgent need for new active biomolecules and enzymes for use in industry and therapy [1]. However, the rate of discovery of new useful compounds has been in decline [2, 3] and because of this there is an interest in investigating previously unexplored ecological niches [4, 5], particularly extreme environments. These environments have provided a useful source of novel biologically active compounds in recent years [1, 6, 7].

Extreme environments are distributed worldwide. These ecosystems were thought to be lifeless as insurmountable extreme physical and chemical barriers to life exhibit. With the advancement of our knowledge, we now see them as yet another niche harbouring “extremophiles” [8]; major categories of extremophiles include halophiles, thermophiles, acidophiles, alkaliphiles, and haloalkaliphiles [6, 9].

The haloalkaliphiles bacteria have attracted a great deal of attention from researchers in this last decade [9]. In 1982, the term haloalkaliphile was used for the first time to describe bacteria that are both halophilic and alkaliphilic [10]. This group of bacteria is able to grow optimally or very well at pH values at or above 10 along with high salinity (up to 25% (w/v) NaCl) [11].

To encounter such harsh conditions, haloalkaliphilic microorganisms have found various physiological strategies to sustain their cell structure and function [12, 13]. These bacteria have widely been identified and studied from the hypersaline environments, soda lakes, solar saltern, salt brines, carbonate springs, and Dead Sea [14]. Their survival obviously indicated the widespread distribution of such organisms in natural saline environments [12, 15].

The interest in haloalkaliphilic microorganisms is due not only to the necessity for understanding the mechanisms of adaptation to multiple stresses and detecting their diversity, but also to their possible application in biotechnology [9].

The present work involved the isolation and characterization of new haloalkalitolerant and haloalkaliphilic bacteria able to produce extremozymes and elaborate natural bioactive compounds effective against pathogenic bacteria and fungi as well. The screening for genes coding for putative antitumor compounds by PCR with three sets of primers was also performed. We have been interested in soils of Algerian Sahara Desert, which is one of the biggest deserts and encompasses one of the most extreme environments worldwide (Sabkha and Chott). However, it is also considered to be one of the less explored parts. Our team has been interested in these magnificent ecosystems for many years and the few studies that have been published have shown great active biomolecules [16–18], biodiversity of interesting new taxa [19–24], and enzymes [25, 26].

## 2. Material and Methods

### 2.1. Sampling and Strains Isolation

**Figure 1:**
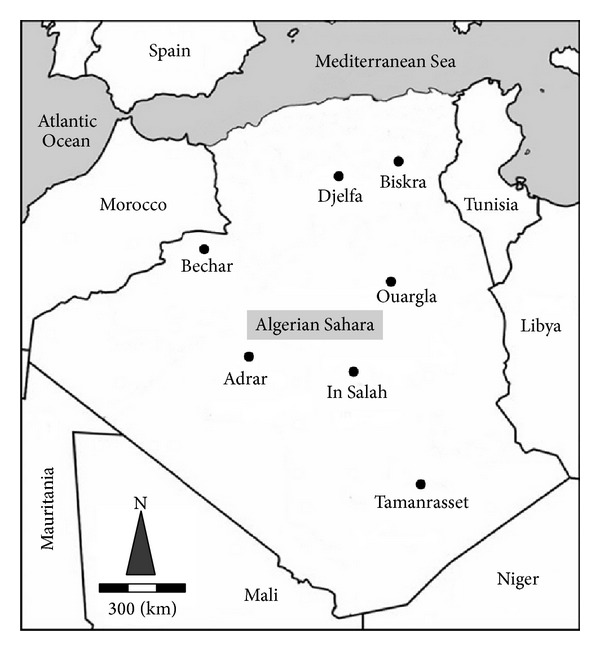
Location of the sampled sites from Algerian Sahara Desert. Djelfa: 35°16′47.5′′N 3°43′25.4′′E; Biskra: 34°11′01.1′′N 6°07′21.8′′E; Ouargla: 33°29′ 28.85 N 5°59′10.52′′ E; Tamanrasset 23°00′30.01′′N 5°13′33.32′′ E; In Salah: 27°11′31.60′′ N 2°27′12.52′′ E; Adrar: 27°44′48.14′′ N 0°16′10.21′′ W; Bechar: 30°51′25.71′′ N 1°59′58.56′′ W.

Samples from different soils (7 sites) of Algeria's Sahara Desert were collected on March 2010 (100–300 g per site in sterile bags) (Figure 1). Most samples were saline and alkaline soils, with an electrical conductivity between 1.4 and 20.2 mS/cm (at 20°C) and pH range of 7.5–9; the temperature varies from 22°C north to 44°C south of the Sahara. One gram from each sample was suspended in 9 ml sterile water (of 0.9%, 10%, and 20% NaCl w/v) and serial dilutions to 10^−4^. For each dilution and for each concentration, soil particles were allowed to sediment; then 0.1 mL of the liquid phase was spread onto the surface of each of the modified International* Streptomyces* Project 2 (ISP2) [27] media agar supplemented with NaCl with respect to the various concentrations of salt used for dilutions (0.9%, 10%, and 20% NaCl w/v) and adjusted to either pH= 7 or pH = 10 by adding 5 M NaOH before autoclaving and spread onto nutrient agar plates. The plates were maintained at constant humidity incubated at either 30°C or 50°C for 15 days. Colonies were picked out and repeatedly restreaked until purity was confirmed. All bacterial culture isolates were stored at 4°C in the same medium used for isolation.

### 2.2. Physiological Growth Parameters

Physiological growth parameters for the thirteen selected strains were determined by agar plate method on modified ISP2 medium depending on the modified parameter. Salinity tolerance was examined for 0, 1, 5, 10, 15, 20, and 25% NaCl w/v. The pH growth range was investigated between pH 5 and 12 at intervals of 1 pH unit. The temperatures tested were 4, 10, 15, 20, 25, 30, 37, 40, 42, 45, 55, and 60°C. Incubation time was one week for* Actinobacteria* and two days for non-*Actinobacteria*.

### 2.3. Molecular Study

#### 2.3.1. DNA Extraction

Total genomic DNA from the different selected bacteria for this study was isolated and purified using Qiagen Blood and Tissue DNA extraction kit (Qiagen, UK). DNA was eluted in Tris-HCL and its quantity and quality were tested using NanoDrop 2000 (Thermo Scientific). DNA was stored at −20°C until use.

#### 2.3.2. Molecular Identification

The amplification of 16S rRNA gene for the selected strains was performed using the universal bacterial primer pairs pA/pH designed by Edwards et al. [28] (Table 1). PCR reactions were performed in final reaction volume of 50 *μ*L containing 1 *μ*L (10–100 ng) of DNA template, 25 *μ*L master mix (Promega, Madison, WI, USA), 1 *μ*L (10 *μ*M) of each primer, and 1 *μ*L of BSA (10 mg/mL) (Promega, Madison, WI, USA). PCR products were analyzed on 1.5% (w/v) agarose (Sigma, UK) in 40 mM Tris-acetate with 1 mM EDTA (TAE) buffer at pH of 8.0 stained with ethidium bromide at 0.5 *μ*g mL^−1^. Bands of the corresponding size were cut out and purified with gel extraction kit (Qiagen; Venlo, Netherlands) as per the manufacturer's instructions.

The nucleotide sequences for the 16S rRNA gene of the different strains were carried out by GATC Biotech (UK). The isolates were identified using the EzTaxon-e server (http://eztaxon-e.ezbiocloud.net/) on the basis of 16S rRNA sequence data [32].

The Molecular Evolutionary Genetics Analysis (MEGA) software, version 4.0.2, was used to assist the phylogenetic analyses and the phylogenetic tree construction [33]. Similar 16S rRNA gene sequences for the studies of the strains were obtained by using Eztaxon [32]. Multiple alignments of data were performed by CLUSTAL W [34]. Evolutionary distances were calculated by using maximum composite likelihood method and are in the units of the number of base substitutions per site [35]. Phylogenetic tree was reconstructed with the neighbour-joining algorithm [36]. Topology of the resultant tree was evaluated by bootstrap analyses of the neighbour-joining dataset, based on 1000 resamplings [37].

The sequences reported in this study have been submitted to NCBI GenBank and the accession numbers are listed in appendices.

### 2.4. Screening

#### 2.4.1. Primers and Molecular Screening

From the thirteen selected strains, six were subjected to molecular screening for genes coding for putative antitumor compounds using three primer sets (Table 1). These strains were chosen on the basis of the presence of nonribosomal peptide synthetases/ polyketide synthases (NRPS/PKS) genes within their genomes (data not published). The first set designed by Decker et al. [29] amplified dNDP-glucose dehydratase genes. The second set was that of Chang and Brady [30] used to screen for biosynthesis of the antitumor substance BE-54017. The final set was used from the study of Ouyang et al. [31] targeting the jadomycin cyclase gene which intervenes in angucycline production.

The PCR mixture included 1-2 *μ*L of genomic DNA, 15 *μ*L master mix (Sigma,UK), 1 *μ*L each of forward and reverse primers (10 *μ*M each) (Sigma, UK), 1 *μ*L of BSA (10 mg/mL) (Promega, Madison, WI, USA), and 6 *μ*L sterile distilled water in a final volume of 25 *μ*L. PCR was performed with Mastercycler pro (Eppendorf). Agarose gels (1% w/v) were photographed after staining with ethidium bromide at 0.5 *μ*g mL^−1^ with a minivisionary imaging system. Sizes of the fragments were estimated using the Fermentas 1 kb Plus DNA ladder (Fermentas, UK).

#### 2.4.2. Antimicrobial Activities Test

Antimicrobial activity was determined by the agar cylinder diffusion method. A 6 mm diameter cylinder was taken from solid cultures and put on preseeded nutrient agar plate of the targeted microorganisms mentioned below. Up to five cylinders of different bacteria per plate were tested. Inhibition zones were expressed as diameter and measured after incubation at 37°C for 24 h for bacteria and at 28°C for 48–72 h for the filamentous fungus and yeasts [38].

Reference strains used in this study were as follows.

Sa:* Staphylococcus aureus* ATCC 25923, Ml:* Micrococcus luteus* ATCC 9341, Ec:* Escherichia coli* ATCC 25922, Pa:* Pseudomonas aeruginosa* ATCC 27853, Ca:* Candida albicans* (clinical isolate, Algerian Central Hospital of Army of Algeria), Foa:* Fusarium oxysporum f.* sp.* albedinis* a filamentous phytopathogenic fungi for date palm (Algerian National Institute for Plant Protection), and Sc:* Saccharomyces cerevisiae*.

#### 2.4.3. Enzymatic Screening

Enzymatic activities “amylolytic, proteolytic (caseinase), and lipolytic” were screened using zone clearance assays. The enzymatic substrate was incorporated to the media, and the strains were restreaked by spots [39]. The tests were conducted with respect to physiological growth parameters of each strain.

## 3. Results

### 3.1. Strains Isolation and Selection

**Figure 2:**
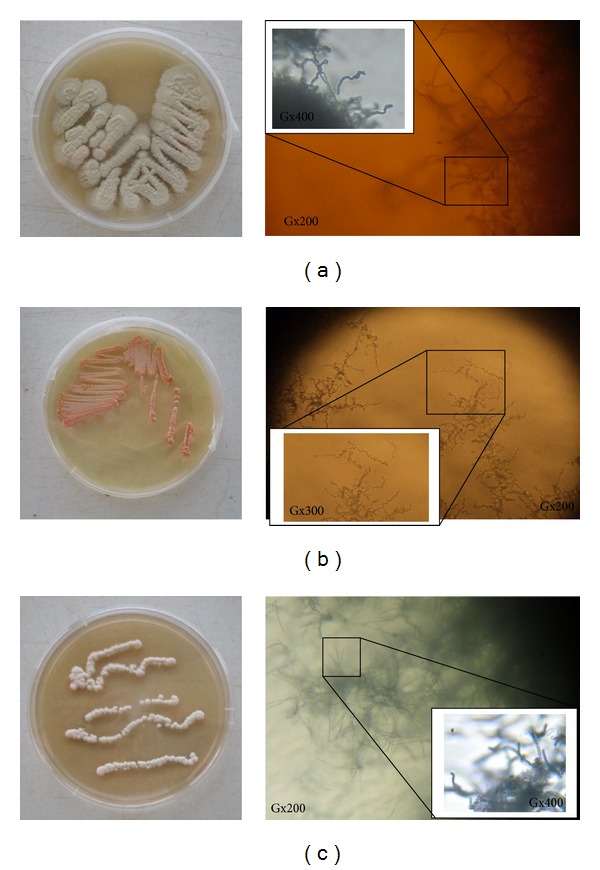
Macroscopic morphology (left) on ISP2 and microscopic filamentous morphology (right) of three strains of this study. (a) Strain IG6: spiral chain of spores on aerial mycelium. (b) Strain Bisk2: nocardioform mycelium. (c) Strain M5A: long straight chains of spores on aerial mycelium.

Isolation plates developed various types of colonies. Sixty to hundred colonies were found per plate in the first dilution for almost all soils, two to ten colonies were observed in the third dilution, and almost nothing in the fourth dilution plates. We have also seen that for the same dilution the number of colonies decreases when the concentration of NaCl increases. One to five colonies which looked less represented were selected from each plate with respect to the haloalkaliphilic character. A total of thirty-nine isolates were distinguished. Amongst these thirty-nine isolates (17 were filamentous, 17 bacilli form, and 5 were cocci form), thirteen strains—eleven with particular morphology (filamentous, which may indicate* Actinobacteria* that are best known for the production of active biomolecules), one bacilli form, and one cocci form—were the subject of our study. The macroscopic and microscopic aspects of three of the thirteen strains are represented in Figure 2. The molecular identification by EzTaxon-e, physiological growth parameters, and enzymatic screening are described in Table 2. The alphabetical strains code used in our study refers to the geographical area origin of isolation; the numerical strains code part is a simple sequential order to differentiate strains.

### 3.2. Physiological Growth Study

All strains could tolerate up to 5% NaCl. Strains Reg1, Ker5, and HHS1 were able to tolerate up to 10%, whereas Bisk4 could tolerate up to 15%. Tag5 growth started at 1% and M5A started growing at 10%; these two strains could grow up to 20% NaCl. Reg1, Ker5, and HHS1 are considered as halotolerant. M5A and Tag5 are considered to be halophilic [40].

All strains except A60 had a versatile range of growth pH (5–12) indicating alkaliphilic growth; A60 (5–9) was only alkalitolerant.

Beside the alkalitolerant character of strain A60, it presented a thermophilic profile (45–60°C). With the exception of strain Bisk4, which may be considered as thermotolerant bacteria since it grows up to 55°C, the other selected bacteria are considered to be mesophiles.

### 3.3. Identification

**Figure 3:**
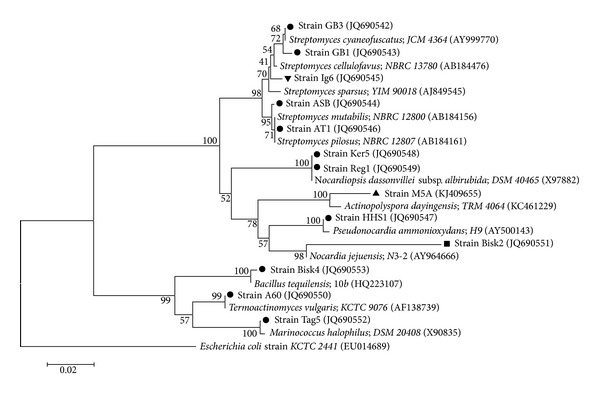
Molecular phylogeny of thirteen selected bacteria and the most related type strains species using partial 16S rRNA sequences. The evolutionary distances were computed using the maximum composite likelihood method and are in the units of the number of base substitutions per site. Tree topology was constructed using MEGA 4.0. Bootstrap values (*n* = 1000 replicates) were indicated at the nodes.* Escherichia coli* KCTC2441 sequence was added as an out group for this tree.

Most isolated strains belonged to the genus* Streptomyces* (AT1, ASB, GB1, Ig6, and GB3). The five* Actinobacteria*, other than* Streptomyces*, were identified as follows:* Reg1* and* Ker5* as two different* Nocardiopsis* sp.,* HHS1* as* Pseudonocardia* sp.,* M5A* as* Actinopolyspora* sp., and Bisk2 as* Nocardia* sp. Bisk2 looks like a new member as it branches out 100% of the time from its nearest relative* Nocardia jejuensis* determined by EzTaxon-e with 95% similarity for the 750 recovered bases. One filamentous strain A60 was identified as* Thermoactinomyces* sp. The bacilli Bisk4 is part of the* Bacillus mojavensis* complex and the cocci Tag5 belonged to the genus* Marinococcus* (Table 2; Figure 3).

### 3.4. Screening for Biotechnological Potential

#### 3.4.1. Screening for Genes Coding for Putative Antitumor Compounds

**Figure 4:**
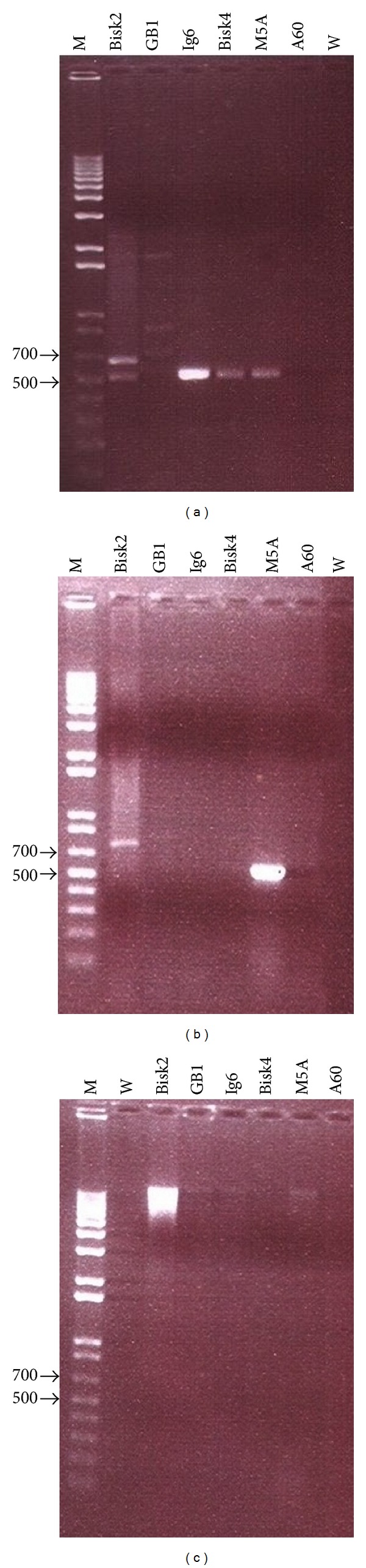
Agarose gel electrophoresis of PCR products from genomic DNA of six strains of the present study with selective fragments amplification range 500–700 bp using primers: (a) Glu1/Glu2, (b) StaDVF/StaDVR, and (c) AuF3/AuF4. M: 1 kb Plus DNA ladder; W: water control.

Glu1/Glu2 primer set had 4/6 positives. High intensity band was registered for the strain Ig6. The primers targeted two different regions for the strain Bisk2. Multiple bands were recovered from the strain GB1 while no one range 500–700 pb. PCR using this primer was negative for the strain A60 (Figure 4(a)). The StaDVF/StaDVR primer set was positive in one strain (Figure 4(b)). The PCR with AuF3/AuF4 primer set was negative for all tested strains (Figure 4(c)).

#### 3.4.2. Antimicrobial Activity

**Figure 5:**
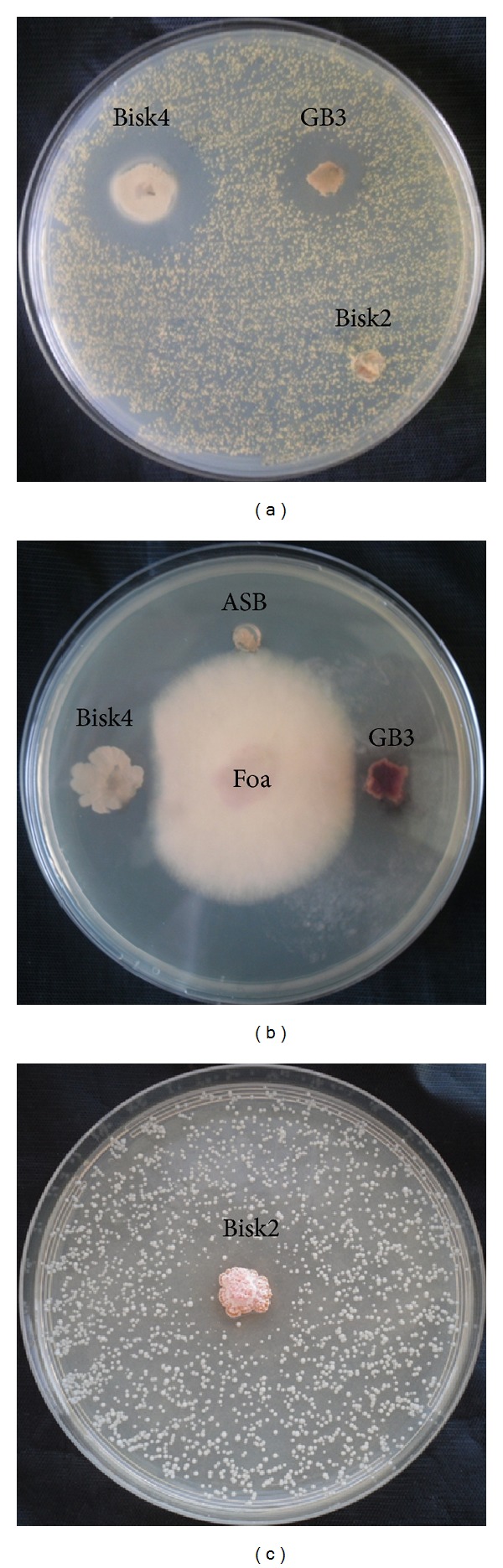
Antimicrobial activity of some strains among the selected strains: (a) antibacterial activity against* Staphylococcus aureus*, (b) antifungal activity against* Fusarium oxysporum f.* sp.* albedinis*, and (c) antifungal activity of the strain* Nocardia* sp. (Bisk2) against the yeast* Saccharomyces cerevisiae.*

The antimicrobial activity of the thirteen selected strains differed between strains (Table 2; Figure 5). Among these, eight showed at least an antimicrobial activity against one of the targeted microorganisms. A highly broad spectrum antimicrobial activity inhibition was seen by the strain* Streptomyces* sp. (GB3). The strain* Bacillus* sp. (Bisk4) had gram positive antibacterial activity and antifungal activity against the filamentous fungi. The strain* Actinopolyspora* sp. (M5A) inhibited the growth of* Micrococcus luteus*. The isolate* Nocardia* sp. (Bisk2) showed a unique and selective activity against the yeast* Saccharomyces cerevisiae* (Figure 5(c)). However, none of the thirteen strains demonstrate specific and unique activity against the gram negative bacteria.

#### 3.4.3. Enzymatic Activity

**Figure 6:**
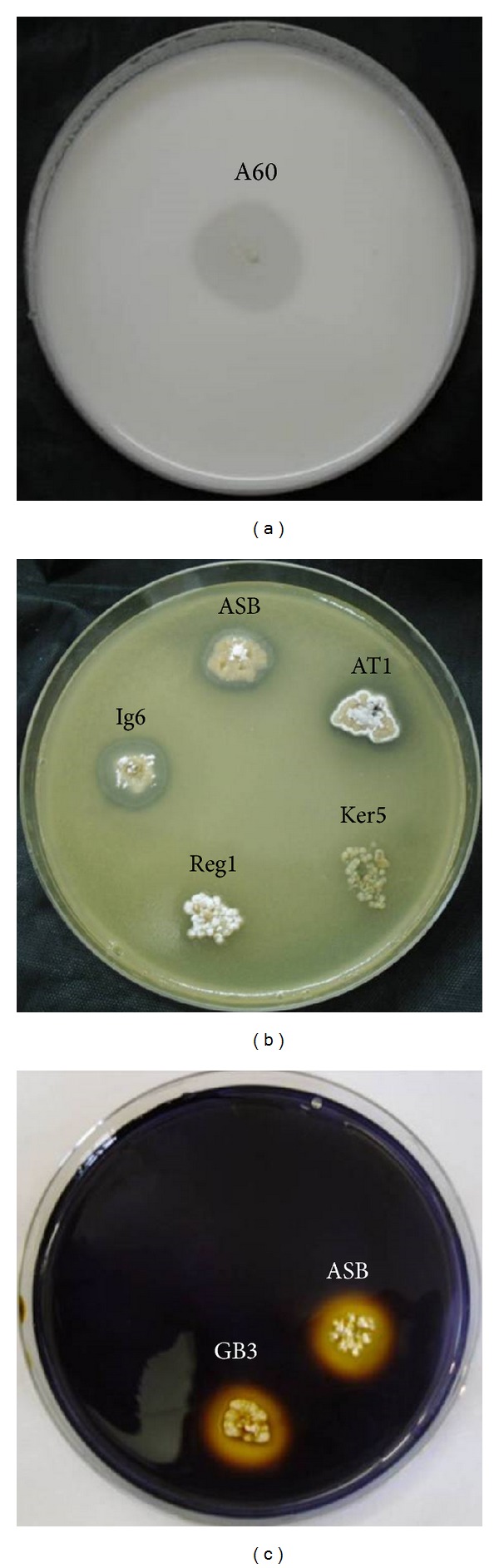
Enzymatic activities of some strains among the selected strains. (a) Proteases (caseinase), (b) lipases, and (c) amylases.

Strains from* Bacillus* and* Streptomyces* were more enzymatically active and possess at least two of the screened enzymes. The strain* Thermoactinomyces* sp. (A60) was able to degrade casein and lipids. Strains Bisk2, TAG5, and HHS1 seemed to have none of these screened enzymes (Figure 6).

## 4. Discussion

In this study we looked at extreme environment of the Algerian Sahara Desert as a source for novel strains possessing interesting bioactive properties. In total, we isolated a collection of thirty-nine haloalkalitolerant and haloalkaliphilic isolates, thirteen of which were selected and screened for genes coding for putative antitumor compounds, as well as screening for antimicrobial and enzymatic activities. All strains were identified using 16S rRNA gene sequencing. This study represents novelty in looking at the relatively understudied areas of Sabkha and Chott and has yielded at least thirteen strains which potentially have antitumorgenic, antimicrobial, and enzymatic properties.

Although often extreme and hostile ecosystems diversity and abundance of bacteria can be low ranging from 10 to 10^4^ UFC/g of soil where the physicochemical parameters are controlling factors [19], the strains retrieved and identified in our study, in particular, of* Actinobacteria* strains, which belong to various taxa, indicate a great diversity. Diversity in environments such as the one in this study has previously been investigated such as in Tunisia [9], China [41], and previously in the Algerian Sahara soils [19, 42], which has revealed that members of these extreme ecosystems are mainly halotolerant or halophilic organisms. Many of the isolated taxa in this study have previously been found in this environment, particularly of the* Actinopolyspora*,* Nocardiopsis*, and* Marinococcus* [9, 41–43]. Despite this their community structure differs both quantitatively and qualitatively for each different ecosystem. This would be due not only to the adaptation to environmental obstacles but also to the geolocalisation [43], the difference of the study protocol (method, media) [41], and the sampling sites [42].

Genome sequencing followed by bioinformatics analysis for some of the already sequenced microorganisms such as* Actinobacteria* and* Bacillus* has revealed the presence of several gene clusters per genome that can produce different molecules [44]. Among the validly described halotolerant and halophilic bacteria, particularly* Actinobacteria*, only few numbers have been subjected to analysis of their bioactive compounds [45]. In addition, many compounds are usually produced in very low amounts (or not at all) under typical laboratory conditions [46]. PCR based methods for specific enzymes activating specific molecules are excellent screening tools for these strains; they would not only indicate the presence of probable genes clusters but also help in biochemical characterisation of the molecules. These methods would help in reducing the number of strains that need to be screened by cultural methods. The PCR based methods not only are limited to genomic DNA but also can be applied for the screening of eDNA that lead to the discovery of new active biomolecules [30]. Screening for potential production of a particular type of biomolecules such as antibiotics and antitumorales, without going through the tedious biochemistry process, is more efficient when the typing protocol is targeting the biosynthesis gene cluster rather than the taxonomic marker genes (e.g., 16S rRNA gene) which often give misleading results [47, 48].

In our study, we have been interested in molecular screening of bioactive genes coding for putative antitumor compounds. The degenerate primers Glu1/Glu2 for the conserved N-terminal sequence of dNDP-glucose 4,6-dehydratase genes have been extensively used to screen out for clusters of active biomolecules with antitumoral activity such as novobiocin [49], enediyne [50], elloramycin [51], sibiromycin [52], ravidomycin, and chrysomycin [53]. The primer set has also been reported in other screening studies for talosins A and B cluster, an antifungal [54], for caprazamycin biosynthesis, an antimycobacterial [55], and more recently we have used this set to screen for amicetin biosynthesis gene cluster, an antibacterial and antiviral agent [56]. The second primer set was designed by Chang and Brady [30] who screened a previously archived soil eDNA cosmid library by PCR using degenerate primers designed to recognize conserved regions in known oxytryptophan dimerization genes (*StaD/RebD/VioB* etc). The oxytryptophan dimerization enzymes were chosen as probes because this enzyme family is used in the biosynthesis of structurally diverse tryptophan dimmers, which have shown an antitumoral activity. Both indolocarbazole biosynthetic gene clusters (e.g., staurosporine, rebeccamycin, K-252a, and AT2433) and violacein biosynthetic gene clusters contain homologous enzymes that carry out the oxidation (*StaO/RebO/VioA*) and subsequent dimerization (*StaD/RebD/VioB*) of tryptophan. One among the six screened strains was positive for the set of the primers, strain M5A. This would signify that the strain M5A could produce tryptophan dimmers compound(s).

The sequencing result followed by blast for the PCR products of M5A using StaDVF/StaDVR primers set (GenBank: KJ560370) has shown 76% homology to the uncultured bacterium clone AR1455 rebeccamycin-like tryptophan dimer gene cluster (GenBank: KF551872) that was studied by Chang and Brady [30], while, for the strains* Streptomyces* sp. Ig6, it has shown a mixed PCR product; we think this is probably due to the presence of multiple variable copies of this gene in this strain.

The different patterns of activity against the targeted microorganisms observed in this study may indicate a variety of the produced active biomolecules. The antimicrobial activity of Bisk2, most closely related to* Nocardia jejuensis* [57], has never been reported to our knowledge. This result encourages us to consider Bisk2 as probably a new member or at least a new strain of* Nocardia*. Genome sequencing, DNA-DNA hybridising, and molecular chemotaxonomy would give more knowledge about its taxonomic position among the* Nocardia* species.

The Sahara Desert is subject to large fluctuations in parameters such as temperature, pH, or salinity. It is populated by communities of organisms with intrinsic genomic heterogeneity for adaptation. The mechanisms of cell adaptation engage several enzymatic processes that may be a source of enzymes that show a higher level of stability and activity over a wider range of conditions. The screened enzymes found in this study (proteases, amylases, and lipases) would be economically valuable since they were screened from such environments and are likely to exhibit rare properties; these extremozymes are of great value to biotechnology industries [7, 58, 59].

## 5. Conclusion

Exploration of biodiversity and biotechnological potential of desert microorganisms has gone several steps forward in recent years. The Sahara Desert is one of the biggest worldwide. It spreads upon several countries of Africa. These countries are among the countries worldwide to have the smallest registration rates of biodiversity in biological databases [60].

In addition to the insights on the biodiversity of Algerian Sahara Desert, to our knowledge, this is the first time to use the molecular screening of these genes coding for putative antitumor compounds to analyse Algerian strains. In this study, we have highlighted the interesting presence of diverse haloalkalitolerant and haloalkaliphilic strains with potential antitumorigenic, bioactive, and other interesting enzymes. Future work will concentrate on more cloning and sequencing for whole clusters, chemical characteristics, identification by application of mass spectrum, and other enzymatic and biochemical techniques that would be more suitable for better determination of the nature of the elaborated compounds produced by the strains identified in this study particularly of* Nocardia* sp. Bisk2,* Actinopolyspora* sp. M5A, and* Streptomyces* sp. Ig6.

## Figures and Tables

**Table 1:** Primers used in this study.

Primers	Gene	Molecules	Reference	PCR programs
pA: AGAGTTTGATCCTGGCTCAG pH: AAGGAGGTGATCCAGCCGCA 1,5 Kb	16S RNA	///////////	[28]	PCR cycles were as follows: 1 cycle at 95°C for 10 min; 35 cycles at 94°C for 1 min, 55°C for 1 min, and 72°C for 2 min; one final cycle at 72°C for 10 min.

Glu1: CSGGSGSSGCSGGSTTCATSGG Glu2: GGGWRCTGGYRSGGSCCGTAGTTG 546 bp	dNDP-Glucose-4,6-dehydratases	//////	[29]	PCR conditions used were 95°C for 4 min; 30 cycles of 95°C for 30 s, 65°C for 30 s, and 68°C for 1.30 min; and a final extension cycle at 68°C for 5 min.

StaDVF: GTSATGMTSCAGTACCTSTACGC StaDVR: YTCVAGCTGRTAGYCSGGRTG. 570 bp	Oxytryptophan dimerization genes (StaD/RebD/VioB) (indolotryptoline biosynthetic gene cluster)	BE-54017, (tryptophan dimmers)	[30]	PCR protocol: 1 cycle of 95°C for 5 min; 7 cycles of 95°C for 30 sec, 65°C for 30 sec with 1°C decrement per cycle to 59°C, and 72°C for 40 sec; 30 cycles of 95°C for 30 sec, 58°C for 30 sec, and 72°C for 40 sec; 1 cycle of 72°C for 7 min; hold at 4°C

AuF3: GAACTGGCCSCGSRTBTT AuR4: CCNGTGTGSARSKTCATSA 600–700 bp	Iadomycin cyclase gene of *Streptomyces venezuelae* ISP5230	Angucycline cyclases Marine sponge	[31]	Optimized PCR conditions were as follows: (1) denaturation at 94°C for 5 min, (2) 30 amplification cycles with denaturation (45 s, 94°C), annealing (60 s, 60°C), and extension (60 s, 72°C), and (3) a final extension at 72°C for 8 min.

**Table 2:** Physiologic characterization, antitumoral genes, enzymatic activity, antimicrobial activity, and most related species of the thirteen selected strains of this study.

Strains	Growth parameters			Antitumor genes			Enzymatic activity			Antimicrobial activity							Most related species
	Salinity interval (% g/L)	pH interval	Temperature interval (°C)	Glu	StaDV	AuF	Proteolytic	Amylolytic	Lipolytic	Sa	Ml	Pa	Ec	Foa	Ca	Sc	
Bisk4	0–15	5–12	20–55	+	−	−	+	+	+	+	+	−	−	+	−	−	*Bacillus tequilensis 10b(T) (Bacillus mojavensis group) *
Bisk2	0–5	5–12	4–42	+	−	−	−	−	−	−	−	−	−	−	−	+	*Nocardia jejuensis N3-2(T) *
M5A	10–20	5–12	30–40	+	+	−	+	+	+	−	+	−	−	−	−	−	*Actinopolyspora dayingensis TRM 4064(T) *
HHS1	0–12	5–12	25–30	N	N	N	−	−	−	−	−	−	−	−	−	−	*Pseudonocardia ammonioxydans H9(T) *
A60	0–5	5–9	45–60	−	−	−	+	−	+	−	−	−	−	−	−	−	*Thermoactinomyces vulgaris KCTC 9076(T) *
AT1	0–10	5–12	20–42	N	N	N	−	+	+	+	+	+	+	−	−	−	*Streptomyces mutabilis NBRC 12800(T) *
Reg1	0–10	5–12	25–42	N	N	N	+	−	−	−	−	−	−	−	−	−	*Nocardiopsis dassonvillei *subsp. *albirubida DSM 40465(T) *
Tag5	1–20	5–12	10–42	N	N	N	−	−	−	−	−	−	−	−	−	−	*Marinococcus halophilus DSM 20408(T) *
Ker5	0–10	5–12	20–42	N	N	N	+	+	−	−	−	−	−	−	−	−	*Nocardiopsis dassonvillei *subsp*. albirubida DSM 40465(T) *
IG6	0–5	5–12	20–40	+	−	−	+	+	+	+	+	−	−	−	+	+	*Streptomyces sparsus YIM 90018(T) *
ASB	0–5	5–12	20–42	N	N	N	+	+	+	+	+	−	−	−	−	−	*Streptomyces pilosus NBRC 12807(T) (Streptomyces pilosus group) *
GB1	0–7	5–12	20–37	−	−	−	+	+	+	−	+	−	−	+	+	+	*Streptomyces celluloflavus NBRC 13780(T) *
GB3	0–7	5–12	20–37	N	N	N	+	+	+	+	+	+	+	+	+	+	*Streptomyces cyaneofuscatus JCM 4364(T) *

+: positive activity, −: negative activity, and N: not tested.

## Appendix

GenBank accession numbers for 16S RNA gene sequences of 13 strains of this study are GB3 (JQ690542), GB1 (JQ690543), ASB (JQ690544), Ig6 (JQ690545), AT1 (JQ690546), HHS1 (JQ690547), Ker5 (JQ690548), Reg1 (JQ690549), A60 (JQ690550), Bisk2 (JQ690551), Tag5 (JQ690552), Bisk4 (JQ690553), and M5A (KJ409655).
